# EQ-5D-5L: a value set for Romania

**DOI:** 10.1007/s10198-022-01481-7

**Published:** 2022-06-10

**Authors:** Elena Olariu, Wael Mohammed, Yemi Oluboyede, Raluca Caplescu, Ileana Gabriela Niculescu-Aron, Marian Sorin Paveliu, Luke Vale

**Affiliations:** 1 Health Economics Group, Population Health Sciences Institute, Newcastle University, Baddiley-Clark Building, Richardson Road, Newcastle upon Tyne, NE2 4AX UK; 2 Department of Statistics and Econometrics, Faculty of Economic Cybernetics, Statistics and Informatics, Bucharest University of Economic Studies, Bucharest, Romania; 3 Department of Pharmacology and Pharmaeconomics, Faculty of General Medicine, Titu Maiorescu University, Bucharest, Romania

**Keywords:** EQ-5D-5L, Value set, Utility, Health technology assessment, I18, I10

## Abstract

**Objective:**

We aimed to develop an EQ-5D-5L value set for Romania.

**Methods:**

In line with the EuroQoL standardized valuation protocol, computer-assisted interviews were conducted face-to-face in a representative sample in Romania (November 2018–November 2019).

Valuation methods included composite time trade-off and discrete choice experiment tasks. Several models were tested, including models that accounted for data censoring, panel structure of the data, heteroscedasticity, conditional logit, and hybrid models. The final model was selected based on logical consistency, theoretical considerations, and use of all available data. We compared our value set with other value sets from Central and Eastern Europe region.

**Results:**

Data from 1493 respondents was used to estimate the value set. A censored hybrid model corrected for heteroscedasticity was selected to represent the value set. The highest decrements in utility were observed for the pain/discomfort dimension (0.375), followed by the mobility dimension (0.293). Health utilities ranged from 1.000 to − 0.323 and 1.3% of the values were negative. The model was corrected with survey weights to better reflect the representativeness of the sample, but the first two coefficients of the self-care dimension stopped being logically consistent. Differences were found between the Romanian, Hungarian and Polish EQ-5D-5L value sets. Good agreement was noted with the Romanian EQ-5D-3L value set, with a swap between pain/discomfort and mobility in ranking of dimensions.

**Conclusion:**

A value set for EQ-5D-5L is now available for Romania. This will push one-step further the development of health technology assessment and encourage more health-related quality-of-life research to be conducted locally.

**Supplementary Information:**

The online version contains supplementary material available at 10.1007/s10198-022-01481-7.

## Introduction

Quality-adjusted life-years (QALYs) are fundamental to Health Technology Assessment (HTA) processes in many countries across the world. Even though much debated in recent years, QALYs are still considered the most rigorous available method to guide healthcare resource allocation decisions [1]. QALYs have the benefit of combining both morbidity and mortality into a single measure allowing transparent and consistent comparisons of different interventions across diseases. To estimate QALYs, duration and health utilities are needed. Health utilities reflect people’s preferences for different health states. They are usually determined in studies conducted in the general population using valuation methods such as visual analogue scale (VAS) or time trade-off (TTO) [2]. In spite of harmonisation and standardization efforts put into valuation studies, health utilities differ considerably from one country to another [3]. These differences have been attributed to differences in either valuation methodologies, sociodemographic backgrounds of respondents, or cultural values [3]. Hence, in order for health utilities to be truly informative for economic evaluations and for QALYs to capture the true impact of morbidity in a certain country, health utilities and value sets should be country specific.

One of the most widely used questionnaires to estimate QALYs is the EQ-5D. The EQ-5D [4] is a simple to use, short, self-reported instrument that measures a person’s current health in five dimensions: mobility, self-care, usual activities, pain/discomfort, and anxiety/depression [5]. It consists of a 5-item descriptive system and a visual analogue scale (EQ VAS). Currently, two versions of the questionnaire are available: the EQ-5D-3L and the EQ-5D-5L. Differences between the two versions include wording changes (standardization of middle levels to moderate in all dimensions for EQ-5D-5L; new descriptor for the most severe level for mobility for EQ-5D-5L) and a change in the number of severity levels in each dimension (three levels of severity for EQ-5D-3L and five, for the EQ-5D-5L [6]). The recall period for EQ-5D-5L is today and each dimension has five response levels: no, slight, moderate, severe, and unable (extreme problems for the pain/discomfort and anxiety/depression dimensions) [6]. The EQ-5D-5L was developed in response to EQ-5D-3L’s ceiling effects and reduced sensitivity to small and medium health changes [6], and has improved psychometric properties when compared with the EQ-5D-3L version [7].

So far, in Romania, the EQ-5D-5L has been used in studies conducted in clinical populations, such as patients with HIV [8], obstructive sleep apnoea [9], cancer [10] or hepatitis C [11]. Nevertheless, the preferred instrument to generate QALYs in Romania is the EQ-5D, just like in many other countries [12]. Currently, the Romanian HTA process uses a scorecard system [13], but local authorities intend to transition to a full HTA process based on cost–utility studies in the near future [14]. Unfortunately, to date, no country-specific value set exists for EQ-5D-5L in Romania, and by default, value sets from other countries, more exactly the United Kingdom, have been used [12]. Value sets can be culturally sensitive [15] and using data from elsewhere to guide healthcare decisions and policies might introduce bias and not represent good value for money on the long run for the Romanian healthcare system [14] Therefore, to encourage the use of national priorities and values, the objective of this study was to develop a country-specific value set for EQ-5D-5L in Romania.

## Methods

This study followed the most recent protocol approved by the EuroQoL research foundation [i.e., the EuroQol Valuation Technique (EQ-VT) version 2.1] [16, 17]. It was developed to allow a parallel estimation of the EQ-5D-3L value set. This manuscript focuses only on the EQ-5D-5L value set, and details on the EQ-5D-3L valuation can be found elsewhere [18].

This study was approved by the Bioethics Committee of Medicines and Medical Devices, Romania (194NP/29.10.2018) and by the Faculty of Medical Sciences Research Ethics Committee, part of Newcastle University's Research Ethics Committee, United Kingdom (Application no. 1430/2069/2018). It was registered with the Romanian National Supervisory Authority for the Processing of Personal Data (Application no. 27512/2017; 28,446/2017).

### Study population

The target population was non-institutionalised adults (18 + years) residing in Romania at the time of the study. Participants were selected using a random-walk technique and next birthday rule from 32 settlements that were randomly selected from all regions of Romania using a three-stage probability sampling procedure stratified by region and settlement size. The sample size was estimated at 1794 participants, so that a representative sample at national level could be achieved. For more details, see Olariu et al. [11, 19].

### Data collection and quality control process

Interviews were face-to-face, computer-assisted and took place in respondents’ homes from November 2018 to November 2019.

Interviewers were trained by the local study team using standardized training materials in a 2-day face-to-face training session in October 2018. Due to five interviewers abandoning the study team early on in the data collection process, another face-to-face training session was organised in June 2019 to recruit more interviewers. In total, 30 interviewers performed data collection. One interviewer was excluded from the study team due to non-compliance with quality criteria.

The interview had five sections: background questions and the EQ-5D-5L, composite time trade-off (cTTO) valuation tasks (five examples, ten EQ-5D-5L tasks, three EQ-5D-3L tasks), discrete choice experiment tasks (DCE) (seven tasks for EQ-5D-5L), the EQ-5D-3L, and a country-specific questionnaire. This structure of the interview had been used before by the US and Hungarian EQ-5D-5L valuation teams [20, 21]. Similar to other data collection tools, the cTTO valuation tasks were developed using 86 EQ-5D-5L health states divided across ten blocks. DCE tasks included 196 pairs of EQ-5D-5L health states, divided across 28 blocks. Each respondent was assigned to one cTTO block and one DCE block [22] (for more details, see Olariu et al. [19].

Interviews’ quality was checked weekly by the local team and every 2 weeks by the EuroQoL research foundation. Interviewers’ performance and compliance to the study’s protocol and guidelines were assessed using the EQ-VT QC software developed by the EuroQoL research foundation [23]. Interviewers were given feedback regarding their performance either by email or by telephone. Interviews were considered of suspect quality if explanations for the wheelchair examples were less than three minutes, if the worse than dead element was not shown in the examples, if the duration of the ten EQ-5D-5L tasks was less than five minutes, and if the worst health state did not have the lowest value or was at least 0.5 lower than the health state with the lowest value [15, 23] [more details here [19, 23].

### Data analysis

All analyses were run using STATA 16 and SPSS 24. Continuous variables were summarised using means, 95% confidence intervals and standard deviations. Categorical variables were reported as frequencies and percentages of all observed levels.

### Exclusion criteria

Prior to modelling, the following exclusion criteria were defined for the cTTO data:

- Participants whose interviews were performed by interviewers excluded from the interviewers’ team.
- Participants whose interviews were performed by interviewers that did not meet the minimum quality criteria as defined by the QC tool.
- Participants whose interviews were performed by interviewers that did not conduct enough interviews (20) to achieve a harmonised learning effect between interviewers [24].
- Participants that did not have any negative values for all cTTO tasks and whose interviews were flagged in the QC report, because the interviewer had not shown the worse than dead element in the example section of the interview.
- Participants that had a positive slope on the regression line between their values and the level sum score of the health states valued or gave the same value to all health states or did not trade time.
- Participants that flagged all ten EQ-5D-5L health states as incorrect on the feedback module.
- Participants with inconsistencies related to the worse health state (55,555) that were not removed after the feedback module.

Additionally, in all models, individual cTTO observations were removed if the respective health state had been flagged by respondents as being incorrect in the feedback module.

Regarding DCE data, participants with suspect patterns in DCE responses were excluded. Suspect patterns in DCE responses were considered those responses that were all the same in all DCE tasks and those that had variations such as ABABABA or BABABAB.

### Model construction

To estimate values for the EQ-5D-5L health states, econometric modelling was used for both cTTO and DCE data. A hybrid modelling approach was used to combine both cTTO and DCE data into a single model [25]. The dependent variable for cTTO data was disutility (one minus the cTTO observed values) and health states were used as explanatory variables. For DCE data, the dependent variable was the binary outcome indicating the respondent’s choice for each pair of EQ-5D-5L states.

We only tested main effects models as the EQ-VT design was optimized for such models [26]. All our models had 20 parameters: four dummies were created for each EQ-5D-5L dimension and level one was used as reference. Our dummies were regular dummies, indicating the loss in utility from level one to that respective level. All models were initially tested with a constant. If the constant was found non-significant at the level of 0.05, it was then supressed.

For cTTO data, we tested Tobit models to account for the censored nature of the data, multilevel models with random intercepts for interviewer and respondent effects, random coefficient models, and heteroskedastic models.

For DCE data, we used a conditional logit model. As DCE valuations are estimated on a latent scale, to allow direct comparisons, we had to anchor them on a scale from 0 (dead) to 1 (full health) by rescaling them using the theta parameter of the best-fitting hybrid model [25].

To make use of all available data, we also tested hybrid models. To test the assumptions of hybrid models, we used scatter plots to plot DCE versus cTTO. If coefficients of the cTTO models were to be proportional to those of DCE models, a line would be observed on the scatter plots. Hence, the assumption of proportionality between DCE and cTTO held true and hybrid models could be estimated [more details on hybrid modelling here [27, 28]].

### Model selection

We used the following criteria to select our final model:

1. Logical consistency of parameters: we only considered models for which coefficients of logically worse health states were lower than coefficients of logically better health states.
2. Significance of parameters and models’ *p* values: we prioritized models that had the maximum number of significant parameters at the level of 0.05 and only considered models that were statistically significant at the level of 0.05.
3. Theoretical considerations:
  models that accounted for the heteroskedastic nature of the data were preferred as the observed variance of the cTTO values increases with the severity of health states [29].
  hybrid models were preferred as they maximise the use of all available data by combining both cTTO and DCE data.
  models that accounted for the censored nature of the data were preferred as by design, the EQ-VT protocol censors observed cTTO values at − 1.
4. Finally, we considered the value range, the ranking of dimensions based on the size of the coefficient for the worst level on each dimension and the correlation between predicted and observed utilities.

The final model was selected based on the consistency of results, correction of heteroscedasticity, accounting for data censoring, and the degree it used all the available data.

### Sensitivity analysis

We performed a sensitivity analysis to test the robustness of our estimated parameters when no exclusion criteria were applied (when no participant was excluded). We also tested the impact of using survey weights on the final model to correct for the disproportionate allocation to strata of our design and potential differences between the sample and the Romanian general population (see electronic supplementary material Annex 1 for more details on survey weights).

### Comparison with other value sets

We compared the observed cTTO values for Romania with those of Hungary and Poland for the 86 health states that were common to all three studies. We used a *z* test to determine the statistical significance of the differences between the observed means for Romania and Hungary and Poland, respectively.

We compared our final EQ-5D-5L value set with the Romanian EQ-5D-3L value set and the Polish and Hungarian EQ-5D-5L value sets. This was done using density plots to observe range of values, modality, or skewness. We also used Bland–Altman plots to check the agreement between the Romanian EQ-5D-3L and EQ-5D-5L utilities of those health states that are comparable across the EQ-5D-3L and EQ-5D-5L (i.e., the matched 243 states).

For the entire data analysis, we used a significance level of *α* = 0.05. We considered correlations to be very strong if correlation coefficients were > 0.9 [30].

## Results

1674 interviews were performed (for response rates and interviews performed in each settlement see Annex 2 of the electronic supplementary material). Of these 1674 interviews, 1493 were used in the analysis (see electronic supplementary material Annex 3 for all interviews excluded). The interviews included in the analysis were performed by 24 interviewers.

**Fig. 1:**
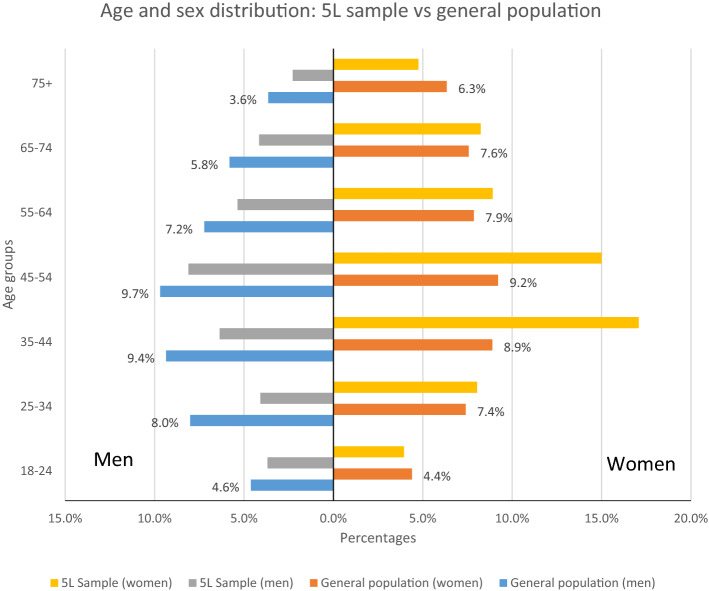
Age and sex distribution in the analysed sample compared with the general population in Romania

The mean age of included respondents was 48.6 years (SD = 16.2) (weighted sample: 47.5 years, SD = 17.8) and the mean EQ VAS was 82.5 (SD = 15.5) (weighted sample: 81.3, SD = 16.6) with the majority of the sample (52.4%) reporting full health (weighted percentage 49.7%) (see Table 1). Overall, in our sample, there were more women (66%) and more people from urban areas (72.8%) than national average statistics. There were also differences in age and sex with respect to the Romanian general population: men were underrepresented in all age groups and women were overrepresented in age groups from 25 to 74 years (see Fig. 1).

**Table 1:** Sociodemographic characteristics of the sample selected for analysis. V6 dataset corresponds to exclusion criteria 1, 2, 3, 4, and 5

Variable	Category	V6 (*n* = 1493)		Weighted V6 (*n* = 1493)		General population
		*n*	%	*n*	%	%
Sex	Female	985	66.0	776	52.0	52
Residence area	Urban	1087	72.8	809	54.2	55.2
Education level	No formal education	6	0.4	11	0.7	2
	Low	177	11.9	232	15.6	36.9
	Medium	747	50.0	795	53.3	45.2
	High	555	37.2	447	29.9	15.9
	No response	8	0.5	8	0.5	
Occupation	Employed	893	59.8	793	53.1	52.1
	Unemployed	32	2.1	49	3.3	3.9
	Retired	370	24.8	396	26.5	26.2
	Stay at home/domestic	105	7.0	132	8.9	7.1
	In education	77	5.2	97	6.5	4.8
	No response	16	1.1	26	1.7	
Income	Below the average	638	42.7	703	47.1	41.4
	Average	264	17.7	248	16.6	30.7
	Above the average	471	31.5	404	27.1	27.9
	No response	120	8.0	137	9.2	
Experience with serious illness	In self	299	20.0	314	21.0	
	In family	680	45.5	662	44.4	N/A
	In caring for others	246	16.5	216	14.5	
Self-rated health using EQ-5D-5L	11,111	782	52.4	743	49.7	N/A
	Any other health state	711	47.6	750	50.3

Interviews lasted on average 47 min (SD = 24). Respondents took on average 83.5 s (SD = 116.4) and 50.5 s (SD = 60) to complete one cTTO and DCE task, respectively. 311 (20.8%) participants provided inconsistent responses regarding the values assigned to cTTO tasks. After the feedback module, 64 respondents reconsidered their choices, thus reducing the number of respondents with logical inconsistencies to 247 (16.5%). Subsequently, the use of the feedback module led to the elimination from analysis of 1,152 cTTO values (7.7% of the total cTTO values). There were only 143 values at zero representing 1% of the valid values assigned in the cTTO tasks^1^ and 679 (4.9%) and 146 (1.1%) values at 0.5 and − 0.5, respectively. 12% of the values assigned by respondents to the health states presented in the valid cTTO tasks were negative and of these 15.9% were values of − 1. 70% of the negative values were assigned to the worst health state (55555). Health states 52455 and 43555 had the second and third highest negative values, but the percentages remained low when compared with 55555 (2.7% and 1.9%, respectively) (see Annex 4).

**Fig. 2:**
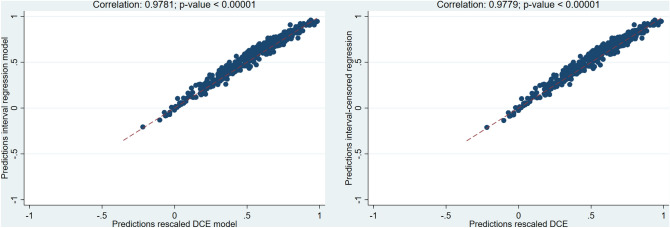
Scatter plots of composite time trade-off (cTTO) model predictions versus rescaled discrete choice experiment (DCE) model predictions

We tested several cTTO and DCE models (see Annex 5 for full list). Table 2 presents the results for those models that had the maximum number of significant and consistent parameters, accounted for heteroscedasticity and/or for the censored nature of the data. All parameters for the selected cTTO models were consistent. The conditional logit model generated two inconsistent parameters at the slight and moderate levels of the self-care domain. However, this was resolved when both cTTO and DCE data were combined using hybrid models, with all hybrid models being consistent. The agreement between cTTO and DCE data was very high as shown by the very strong correlations (> 0.9) between the predictions of the cTTO models and rescaled DCE model (see Fig. 2).

**Table 2:**
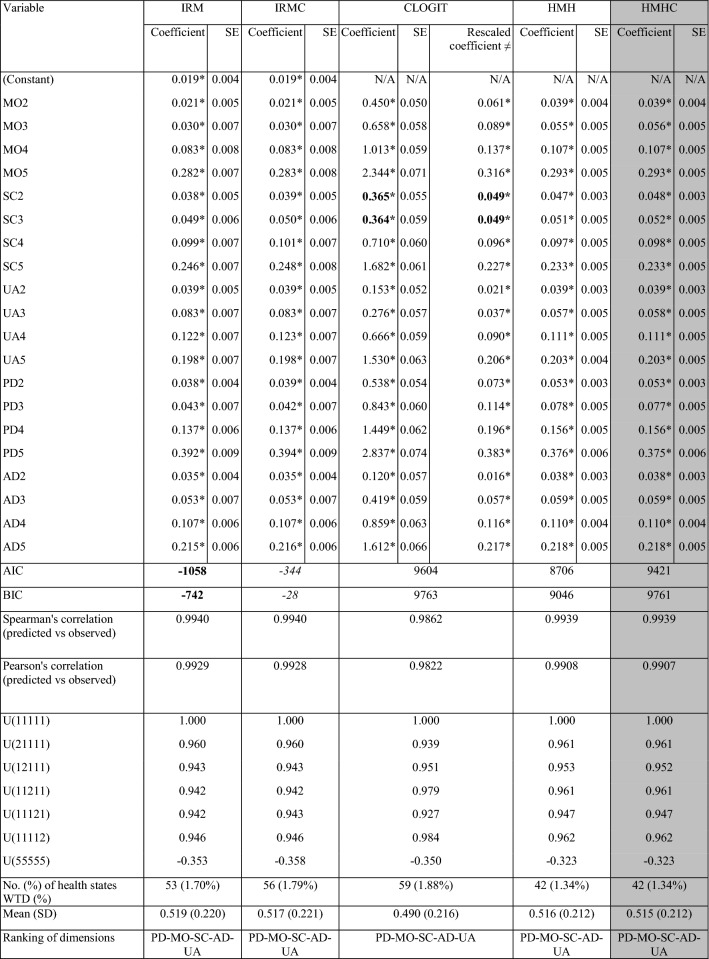
Estimation results for selected cTTO, DCE, and hybrid models

*IRM* interval regression model, *IRMC* interval regression model censored at – 1, *CLOGIT* conditional logit model, *HMH* hybrid model heteroskedastic without constant, *HMHC* hybrid model heteroskedastic without constant censored at – 1, *MO* mobility; *SC* self-care; *UA* usual activities, *PD* pain discomfort, *AD* anxiety depression, *U* utility, *AIC* Akaike information criteria, *BIC* Bayesian information criterion, *SD* standard deviation, *WTD* worse than dead

*p value < 0.05; values in bold—best performance for the indicator; values in italics—second best performance for the indicator; shaded columns—final model chosen; ≠ theta value 7.410

The two cTTO models performed similarly in terms of range of values, ranking of dimensions, and correlation coefficients (see Table 2). The censored model had a slightly higher number of negative values than the uncensored model. In line with our model selection criteria, our preferred cTTO model was the censored interval regression model (IRMC). The two hybrid models that met our selection criteria had almost identical performance in terms of range of values, ranking of dimensions, correlation coefficients, and number of negative health states (see Table 2). Of the two hybrid models, we preferred the censored hybrid model heteroskedastic (HMHC) given the censored nature of the data. Finally, we chose the HMHC model (the Romanian EQ-5D-5L model) over the IRMC model as the hybrid model used all available data in line with our theoretical considerations and aims (see Annex 6 for full details of the model).

### Sensitivity analysis

When our final model was run on the full dataset with no exclusions applied (dataset V1—see Annex 7 for sociodemographic characteristics and Annex 8 for full model), it generated a value set with fewer negative values and a lower range of values (see Table 3). Overall, the performance of the model decreased when interviews that did not meet the quality criteria standards were kept in the sample. When the model was run on the weighted sample (weighted V6 dataset), it generated two inconsistent parameters at the slight and moderate levels of the self-care dimension (see Table 3 and Annex 9 for full model).

**Table 3:**
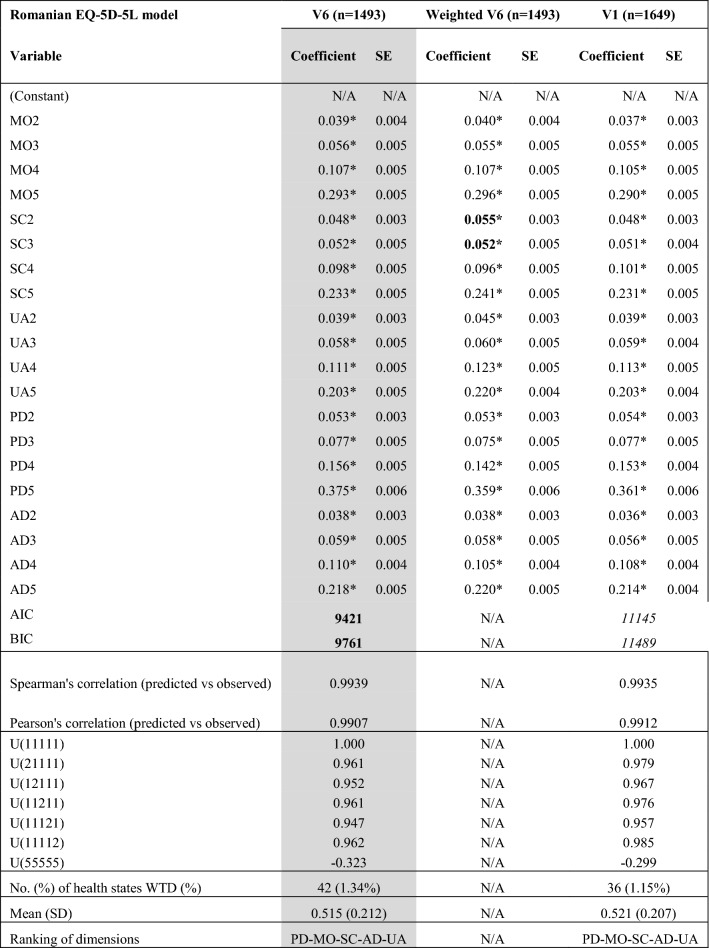
Sensitivity analysis results

*MO* mobility, *SC* self-care, *UA* usual activities, *PD* pain discomfort, *AD* anxiety depression, *U* utility, *AIC* Akaike information criteria, *BIC* Bayesian information criterion, *SD* standard deviation, *WTD* worse than dead, *V1* the dataset that includes all interviews performed with the exception of those interviews performed by interviewers that were subsequently excluded from the interviewers’ team due to quality control issues; *V6* the dataset that includes those interviews that were valid after all exclusion criteria have been applied

*p value < 0.05; values in bold—best performance for the indicator; values in italics—second best performance for the indicator; shaded columns—final model chosen

Finally, we compared the predictions of the final model run on dataset V1 and V6 regarding the mean observed cTTO values and the model performed well in all cases (see Annex 10).

### Comparison with other value sets

**Fig. 3:**
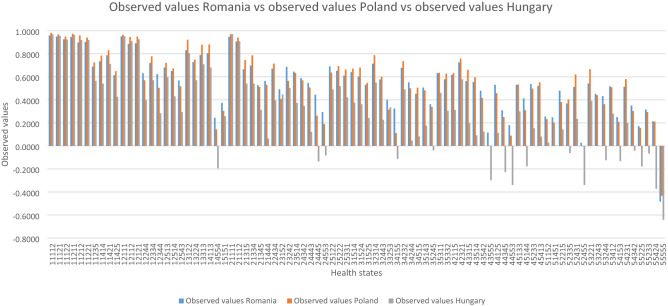
Observed composite time trade-off (cTTO) values for the 86 EQ-5D-5L health states common to the Hungarian, Romanian, and Polish EQ-5D-5L valuation studies

First, we compared our observed cTTO values with the observed cTTO values from the Polish and Hungarian valuation studies for all 86 health states that were common to all three studies (see Fig. 3). We tested the statistical significance between the observed mean differences, and we found that all differences between the observed Romanian and Hungarian cTTO values were statistically significant, with the exception of the differences observed for four health states 11121, 11122, 12111, and 21112. In 91.4% of the cases, the Romanian observed values were higher than the Hungarian observed values with differences ranging from 0.011 to 0.59 (see Annex 11). Regarding the Polish observed values, only 72% of the observed differences were statistically significant: of these, the Romanian observed values were lower than the Polish ones in 61.3% of the cases.

**Fig. 4:**
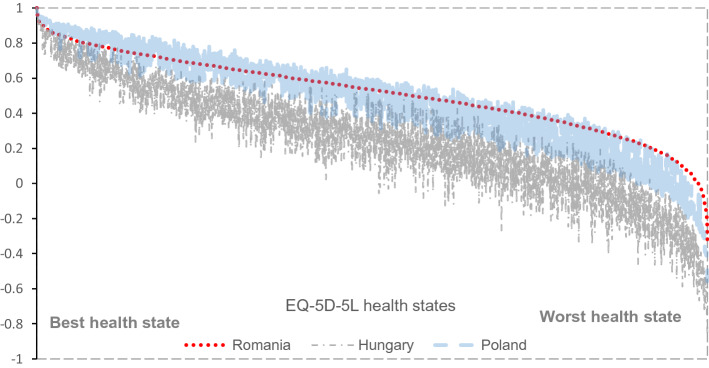
Comparison between the EQ-5D-5L value sets for Hungary, Poland, and Romania

We then compared all our estimated values with the Hungarian and the Polish EQ-5D-5L value sets. Similar to the observed cTTO values, the Hungarian EQ-5D-5L value set was consistently lower than the Romanian EQ-5D-5L value set. Regarding the Polish EQ-5D-5L value set, the values of the Romanian EQ-5D-5L value set were lower than the Polish ones for mild states, but as severity increased, the Romanian values became higher than the Polish ones (see Fig. 4). Also, Romanians assigned the highest value to the worst health state (55,555) (− 0.323 versus -0.590 for Poland [31] versus − 0.848 for Hungary [21] from all the three countries that we compared. Finally, the importance order of the five dimensions of the questionnaire was different from one country to another: Romanians, just like the Polish, placed most weight on the pain/discomfort dimension, whereas Hungarians ranked mobility first. The anxiety/depression dimension came fourth for all three countries (dimension order for Hungary: mobility, pain/discomfort, self-care, anxiety/depression, usual activities; dimension order for Poland: pain/discomfort, mobility, self-care, anxiety/depression, and usual activities).

**Fig. 5:**
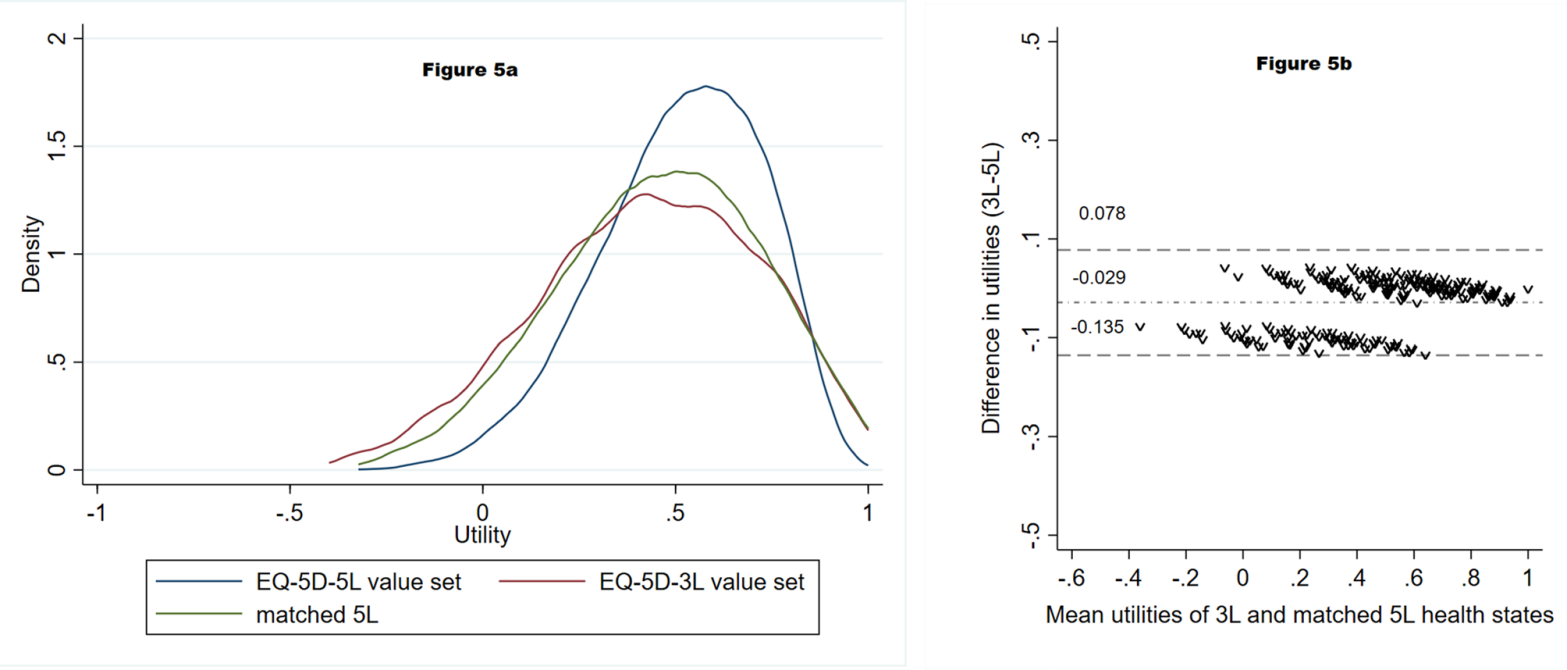
Comparison between the Romanian EQ-5D-3L value set and the Romanian EQ-5D-5L value set using a Kernel density plot (**a**) and a Bland–Altman plot (**b**)

Finally, we compared our EQ-5D-5L value set with the Romanian EQ-5D-3L value set [18]. The Kernel density plot showed a unimodal right-skewed distribution for the EQ-5D-5L value set, whereas the EQ-5D-3L distribution had a few clusters. More EQ-5D-3L values were concentrated at both ends of the utility scale than in case of the EQ-5D-5L distribution, differences being starker between the two for the left-end of the utility scale (see Fig. 5a). The Bland–Altman plot (Fig. 5b) showed a good agreement across the severity scale between the EQ-5D-3L values and the EQ-5D-5L values of the matched health states, with none of the differences falling outside of the ± 2 SD range. The EQ-5D-3L value set had more negative values than the EQ-5D-5L and the importance of dimensions changed slightly: the most important EQ-5D-3L dimension (MO) became second in the case of EQ-5D-5L and the second most important EQ-5D-3L dimension (PD) became first in the case of EQ-5D-5L. The order of the remaining dimensions stayed the same (see Annex 12).

## Discussion

In this study, we estimated a Romanian value set for the EQ-5D-5L. We did this according to the latest EQ-VT protocol approved by the EuroQoL research foundation and following best practice in the field. We used a main effects model that combined both cTTO and DCE data and accounted for heteroscedasticity and censored data to estimate our value set. This modelling approach reflects best practice and current analytical advances in the field [17]. We chose our model from several candidate models based on maximising the number of consistent and significant parameters and theoretical considerations such as heteroscedasticity correction, data censoring, and maximum use of the collected data.

We opted for a censored hybrid model corrected for heteroscedasticity as our final model. We based our decision on the strong [30]agreement between the cTTO and DCE data, which supported the use of single estimation [25] considering cTTO and DCE responses to be stemming from the same unique utility function [25]. The simultaneous use of the two elicitation methods allowed us to get a better picture of the “true” preferences of our respondents, as DCE answers can improve our understanding of cTTO answers [25]. Additionally, it improved our precision in estimating the parameters of our model as reflected by the lower standard errors for all coefficients of our final model when compared with the rest of the models. Finally, it allowed us to maximise the use of all available data. Nevertheless, others might question our decision to use a hybrid modelling approach in determining our value set. This is, because, in spite of the benefits of hybrid modelling, to date, there is no agreement on which modelling strategy might be the best in estimating value sets [31]: methods that use cTTO data only [21, 32], DCE scoring algorithms anchored on TTO data; [33, 34] or methods that use both type of data [31, 35–39] are consistently reported in the literature. Additionally, some argue that there is not yet available a robust theoretical justification to combine the two elicitation methods as they represent two very distinct valuation methods [40].

We compared our EQ-5D-5L value set with the Polish and Hungarian EQ-5D-5L value sets. We chose these two countries as, in our opinion, Central and Eastern European (CEE) countries have a certain degree of similarity in terms of history and culture. Also, they were the only two countries in the region that had, at the time of writing this manuscript, an EQ-5D-5L value set (at that time, Slovenia only had an EQ-5D-3L value set[41]). Nevertheless, differences were noted between the three value sets in terms of values assigned to the worst health state and the relative importance of the five EQ-5D-5L dimensions. The anxiety/depression dimension was ranked last but one in all three CEE countries. This is in contrast with other Western European countries where it ranked second [32, 35] or even first [42]. Greater stigma and lower awareness about mental health problems in Romania and other CEE countries [43, 44] might be behind this finding. Finally, modelling approaches in arriving to the final value set differed from one country to another: Hungary used only cTTO data for [32] their model and both Poland and Romania used cTTO and DCE data for their final model. Hence, all these differences underline once more the importance of having a national value set for EQ-5D questionnaires.

We did not compare our value set with the English EQ-5D-5L value set in spite of it being the default option used locally in Romania, especially by researchers [45–47]. We decided this given the ongoing study to develop a new EQ-5D-5L value set for the UK [48] and the criticisim the current EQ-5D-5L value set for England has received [49].

Our EQ-5D-5L value set was fairly similar to the EQ-5D-3L value set in terms of range of values, value for the worst health states, and the ranking of the last three dimensions. The EQ-5D-5L value set had fewer negative values than the EQ-5D-3L value set, but this is in line with the other studies’ results that used the same methodology as ours [21]. Nevertheless, we recommend the use of the EQ-5D-5L descriptive system in Romania due to its improved psychometric properties [6], reserving the EQ-5D-3L for historical cross-country comparisons or clinical trials that might deem its use more appropriate in their clinical population. Regarding the choice between value sets, national value sets should always be preferred, but other factors should also be considered such as the context in which the results might be used, their research application, and the decisions they might influence [50].

Our study has several limitations. First, changes in the members of the local study team could have affected how the feedback and second session of training were provided to the team of interviewers in spite of the team’s efforts to standardize them. Also, our team of interviewers changed its members several times throughout the study making it difficult to keep the team small and motivated enough to ensure a low variability in how interviews were performed. Nevertheless, new comers were always trained and interviewer bias should have been reduced through the regular use of the QC tool. Second, our sample differed from the Romanian general population in terms of age, sex, and place of residence: men were underrepresented in all age categories and places of residence (only 29% of the interviewed men lived in rural areas). One reason why men are underrepresented in our sample, especially in rural areas, might be the temporary migration for work that has been booming in Romania in the past 2 decades. The seasonal agricultural market from abroad attracts each year many Romanian rural workers, especially men [51]. To see whether these differences had a meaningful impact, we checked whether the observed cTTO values differed according to age, age groups, sex, and place of residence. In line with previous literature [52, 53], we found differences between people from rural and urban areas and between different age groups when they valued health states. When stratified by health state or severity, there was no clear pattern of differences in health state values between groups, with significance achieved in less than 30% of the cases when stratified by severity and less than 15% when stratified by health state. Nevertheless, we decided to adjust our final model with survey weights to account for these differences. Unfortunately, most probably due to the added extra complexity, our final model stopped being consistent when survey weights were introduced.

## Conclusion

In this study, we developed a Romanian value set for the EQ-5D-5L using both cTTO and DCE data. The availability of a national value set for the EQ-5D is a landmark event in the development of HTA in Romania and potentially in the CEE region. It will not only encourage the development of a more locally data-driven HTA process, but also promote cross-country comparisons and collaborations in the CEE region.

## Supplementary Information

Below is the link to the electronic supplementary material.

Supplementary file1 (XLSX 101 KB)

Supplementary file2 (DOCX 103 KB)

## Data Availability

The datasets analysed during the current study are available from the corresponding author on reasonable request.

## Footnotes

1 Valid cTTO values—values assigned to health states that were not marked as being incorrect by the respondent on the feedback module.
