# Supplementary material for: EQ-5D-5L: a value set for Romania
Source: Eur J Health Econ. 2022 Jun 10;24(3):399–412. doi: 10.1007/s10198-022-01481-7 (PMC10060331; doi:10.1007/s10198-022-01481-7)
Supplement: Supplementary file 2 — Supplementary file2 (DOCX 103 KB) [file 10198_2022_1481_MOESM2_ESM.docx]

Journal: The European Journal of Health Economics

EQ-5D-5L: a value set for Romania

Elena Olariu^1*^, Wael Mohammed^1^, Yemi Oluboyede^1^, Raluca Caplescu^2^, Ileana Gabriela Niculescu-Aron^2^, Marian Sorin Paveliu^3^, Luke Vale^1^

^1^ Health Economics Group, Population Health Sciences Institute, Newcastle University, Newcastle upon Tyne, United Kingdom

^2^ Department of Statistics and Econometrics, Faculty of Economic Cybernetics, Statistics and Informatics, Bucharest University of Economic Studies, Bucharest, Romania
^3^ Department of Pharmacology and Pharmaeconomics, Faculty of General Medicine, Titu Maiorescu University, Bucharest, Romania

* Corresponding author

Address for correspondence: Population Health Sciences Institute, Baddiley-Clark Building, Richardson Road, Newcastle upon Tyne, NE2 4AX, United Kingdom. Email: elena.olariu@newcastle.ac.uk. Telephone: +44 (0) 191 208 6949.

# Electronic supplementary material

Annex 1 Method for estimating the survey weights used to adjust the representativeness of the sample used to build an EQ-5D-5L value set in Romania

Our survey weights were calculated as the product of design weights, non-response weights and post-stratification weights. Design weights accounted for the disproportionate allocation to strata, being estimated as the inverse of the respondents’ probability of selection for each of the stages of the survey. Non-response weights were calculated as the percentage of people responding to the survey in each settlement. As differences were found between the sample and the Romanian general population in terms of age, sex and place of residence, post-stratification weights were determined using the same variables that were used to create poststrata (raking method). The 2011 Romanian census was used to create population control totals for each poststratum.

Annex 2 Interviews performed in each settlement and refusal rates

| **Strata No.** | **Strata label** | **County** | **Settlement** | **Target interviews** | **Actual interviews** | **Refusal rates (%)** |
| --- | --- | --- | --- | --- | --- | --- |
| 1 | Cities > 1 mil inh | Municipiul Bucureşti | Municipiul Bucureşti | 382 | 355 | 51.3 |
| 2 | 160.000 inh. ≤ cities < 1 mil inh | Judeţul Prahova | Municipiul Ploieşti | 74 | 75 | 39.84 |
|  |  | Judeţul Dolj | Municipiul Craiova | 74 | 74 | 34.51 |
|  |  | Judeţul Timiş | Municipiul Timişoara | 74 | 76 | 38.84 |
|  |  | Judeţul Cluj | Municipiul Cluj-Napoca | 74 | 58 | 36.21 |
| 3 | 50.000 inh. ≤ cities <160.000 inh. | Judeţul Mehedinţi | Municipiul Drobeta-Turnu Severin | 67 | 67 | 37.96 |
|  |  | Judeţul Sibiu | Municipiul Sibiu | 67 | 68 | 34.95 |
|  |  | Judeţul Satu Mare | Municipiul Satu Mare | 67 | 74 | 27.96 |
|  |  | Judeţul Buzău | Municipiul Buzău | 67 | 67 | 47.24 |
| 4 | South: cities < 50.000 inh. | Judeţul Prahova | Oraş Plopeni | 44 | 49 | 29.03 |
| 5 | South-West: cities < 50.000 inh | Judeţul Dolj | Municipiul Băileşti | 44 | 44 | 16.98 |
| 6 | West: cities < 50.000 inh | Judeţul Hunedoara | Oraş Petrila | 44 | 44 | 16.98 |
| 7 | Centre: cities < 50.000 inh | Judeţul Alba | Oraş Teiuş | 44 | 44 | 13.73 |
| 8 | North-West: cities < 50.000 inh | Judeţul Bistriţa-Năsăud | Oraş Năsăud | 44 | 44 | 10.20 |
| 9 | North-East: cities < 50.000 inh | Judeţul Suceava | Municipiul Fălticeni | 44 | 48 | 18.64 |
| 10 | South-East: cities < 50.000 inh | Judeţul Constanţa | Oraş Ovidiu | 44 | 47 | 21.43 |
| 11 | Bucharest-Ilfov: cities < 50.000 inh | Judeţul Ilfov | Oraş Otopeni | 44 | 0 | N/A* |
| 12 | South: rural settlements | Judeţul Călăraşi | Mitreni | 33 | 5 | 0 |
|  |  | Judeţul Giurgiu | Clejani | 33 | 26 | 13.16 |
| 13 | South-West: rural settlements | Judeţul Gorj | Cătunele | 33 | 33 | 23.26 |
|  |  | Judeţul Dolj | Giubega | 33 | 33 | 13.16 |
| 14 | West: rural settlements | Judeţul Caraş-Severin | Dognecea | 33 | 57 | 11.11** |
|  |  | Judeţul Timiş | Pietroasa | 33 | 33 | 8.33 |
| 15 | Centre: rural settlements | Judeţul Mureş | Rîciu | 33 | 33 | 10.81 |
|  |  | Judeţul Braşov | Hârseni | 33 | 0 | N/A* |
| 16 | North-West: rural settlements | Judeţul Sălaj | Ip | 33 | 39 | 11.36 |
|  |  | Judeţul Cluj | Ciucea | 33 | 33 | 8.33 |
| 17 | North-East: rural settlements | Judeţul Iaşi | Şcheia | 33 | 34 | 8.11 |
|  |  | Judeţul Neamţ | Bozieni | 33 | 32 | 11.11 |
| 18 | South-East: rural settlements | Judeţul Tulcea | Slava Cercheză | 33 | 33 | 19.51 |
|  |  | Judeţul Galaţi | Priponeşti | 33 | 33 | 10.81 |
| 19 | Bucharest-Ilfov: rural settlements | Judeţul Ilfov | Cernica | 33 | 16 | 72.88 |
| Total |  |  |  | 1793 | 1674 |  |

* No interviews were performed in Otopeni and Hârseni, so refusal rates were not estimated.

** Refusal rates were calculated after the exclusion of the 25 interviews performed by the interviewer that was excluded from the interviewers’ team.

Annex 3 Interviews excluded from the sample used to build the Romanian EQ-5D-5L value set and reasons for exclusion

181 interviews were excluded from the sample that was used to build the Romanian EQ-5D-5L value set due to the following reasons (criteria might overlap):

1. 25 interviews were performed by interviewers that were excluded from the interviewers’ team due to quality concerns
2. 81 interviews were excluded due to either not meeting the minimum quality criteria (26), and/or being performed by interviewers that performed less than 20 interviews (52), and/or not having any negative values for all composite time trade-off (cTTO) tasks and having been flagged because the interviewer had not shown the worse than death element in the example section of the interview (21)
3. 15 interviews were excluded because the respondent did not trade any years (four), gave the same value to all health states (one) or had a positive slope on the regression line between their values and the misery index of the health states valued (ten)
4. 32 interviews were excluded because they had a suspect pattern in their responses to the discrete choice experiment (DCE) tasks
5. 44 interviews were excluded because inconsistencies regarding health state 55555 were not removed after the feedback module.

Annex 4 Observed summary statistics for the 86 EQ-5D-5L health states

| **Health state** | **n** | **Mean** | **SD** | **Median** | **25th**  **percentile** | **75th**  **percentile** | **Negative**  **values** |
| --- | --- | --- | --- | --- | --- | --- | --- |
| 11112 | 302 | 0.958 | 0.053 | 0.95 | 0.95 | 1 | 0 |
| 11121 | 279 | 0.949 | 0.083 | 0.95 | 0.95 | 1 | 0.36 |
| 11122 | 144 | 0.926 | 0.074 | 0.95 | 0.9 | 0.95 | 0 |
| 11211 | 295 | 0.946 | 0.069 | 0.95 | 0.95 | 1 | 0 |
| 11212 | 134 | 0.899 | 0.076 | 0.9 | 0.85 | 0.95 | 0 |
| 11221 | 142 | 0.903 | 0.078 | 0.9 | 0.85 | 0.95 | 0 |
| 11235 | 142 | 0.688 | 0.181 | 0.7 | 0.6 | 0.8 | 0.7 |
| 11414 | 137 | 0.734 | 0.177 | 0.75 | 0.7 | 0.85 | 0.73 |
| 11421 | 133 | 0.786 | 0.146 | 0.8 | 0.75 | 0.9 | 0 |
| 11425 | 139 | 0.614 | 0.257 | 0.65 | 0.45 | 0.8 | 2.16 |
| 12111 | 300 | 0.95 | 0.058 | 0.95 | 0.9 | 1 | 0 |
| 12112 | 132 | 0.885 | 0.165 | 0.9 | 0.85 | 0.95 | 0.76 |
| 12121 | 138 | 0.891 | 0.099 | 0.9 | 0.85 | 0.95 | 0 |
| 12244 | 129 | 0.631 | 0.22 | 0.65 | 0.55 | 0.75 | 0.78 |
| 12334 | 142 | 0.72 | 0.146 | 0.75 | 0.65 | 0.85 | 0 |
| 12344 | 121 | 0.624 | 0.186 | 0.65 | 0.5 | 0.75 | 0 |
| 12513 | 125 | 0.68 | 0.165 | 0.7 | 0.6 | 0.8 | 0 |
| 12514 | 143 | 0.65 | 0.215 | 0.65 | 0.55 | 0.8 | 0.7 |
| 12543 | 137 | 0.568 | 0.205 | 0.6 | 0.4 | 0.75 | 0.73 |
| 13122 | 149 | 0.831 | 0.105 | 0.85 | 0.8 | 0.9 | 0 |
| 13224 | 138 | 0.728 | 0.144 | 0.75 | 0.65 | 0.8 | 0 |
| 13313 | 129 | 0.789 | 0.127 | 0.8 | 0.75 | 0.9 | 0 |
| 14113 | 145 | 0.805 | 0.104 | 0.8 | 0.75 | 0.9 | 0 |
| 14554 | 125 | 0.244 | 0.374 | 0.3 | 0.1 | 0.45 | 13.6 |
| 15151 | 144 | 0.374 | 0.396 | 0.45 | 0.25 | 0.6 | 10.42 |
| 21111 | 274 | 0.948 | 0.096 | 0.95 | 0.95 | 1 | 0.36 |
| 21112 | 126 | 0.906 | 0.093 | 0.9 | 0.9 | 0.95 | 0 |
| 21315 | 146 | 0.664 | 0.256 | 0.7 | 0.6 | 0.85 | 3.42 |
| 21334 | 137 | 0.697 | 0.157 | 0.7 | 0.6 | 0.8 | 0 |
| 21345 | 130 | 0.527 | 0.281 | 0.6 | 0.4 | 0.7 | 3.08 |
| 21444 | 128 | 0.564 | 0.226 | 0.6 | 0.45 | 0.7 | 0.78 |
| 22434 | 132 | 0.67 | 0.159 | 0.7 | 0.575 | 0.8 | 0 |
| 23152 | 129 | 0.492 | 0.267 | 0.5 | 0.35 | 0.7 | 1.55 |
| 23242 | 141 | 0.687 | 0.193 | 0.7 | 0.6 | 0.8 | 0.71 |
| 23514 | 132 | 0.645 | 0.175 | 0.65 | 0.55 | 0.8 | 0 |
| 24342 | 133 | 0.588 | 0.251 | 0.65 | 0.5 | 0.75 | 1.5 |
| 24443 | 132 | 0.547 | 0.205 | 0.6 | 0.4 | 0.7 | 0.76 |
| 24445 | 149 | 0.444 | 0.258 | 0.45 | 0.3 | 0.6 | 4.03 |
| 24553 | 136 | 0.294 | 0.368 | 0.35 | 0.2 | 0.5 | 11.76 |
| 25122 | 132 | 0.691 | 0.17 | 0.7 | 0.6 | 0.8 | 0 |
| 25222 | 140 | 0.652 | 0.291 | 0.7 | 0.6 | 0.8 | 3.57 |
| 25331 | 131 | 0.61 | 0.311 | 0.65 | 0.5 | 0.8 | 3.82 |
| 31514 | 139 | 0.64 | 0.181 | 0.65 | 0.5 | 0.8 | 0.72 |
| 31524 | 131 | 0.603 | 0.229 | 0.65 | 0.5 | 0.75 | 1.53 |
| 31525 | 130 | 0.531 | 0.263 | 0.55 | 0.45 | 0.7 | 3.85 |
| 32314 | 136 | 0.714 | 0.16 | 0.75 | 0.65 | 0.85 | 0 |
| 32443 | 138 | 0.576 | 0.183 | 0.6 | 0.45 | 0.7 | 0 |
| 33253 | 141 | 0.401 | 0.319 | 0.4 | 0.25 | 0.6 | 5.67 |
| 34155 | 145 | 0.324 | 0.324 | 0.35 | 0.2 | 0.55 | 10.34 |
| 34232 | 141 | 0.678 | 0.173 | 0.7 | 0.6 | 0.8 | 0 |
| 34244 | 131 | 0.552 | 0.229 | 0.6 | 0.4 | 0.7 | 0.76 |
| 34515 | 140 | 0.453 | 0.301 | 0.45 | 0.35 | 0.675 | 5.71 |
| 35143 | 134 | 0.507 | 0.322 | 0.55 | 0.4 | 0.7 | 3.73 |
| 35245 | 142 | 0.362 | 0.355 | 0.4 | 0.25 | 0.6 | 9.15 |
| 35311 | 142 | 0.632 | 0.321 | 0.7 | 0.55 | 0.8 | 3.52 |
| 35332 | 138 | 0.578 | 0.261 | 0.65 | 0.5 | 0.75 | 2.9 |
| 42115 | 138 | 0.617 | 0.238 | 0.65 | 0.45 | 0.8 | 1.45 |
| 42321 | 136 | 0.725 | 0.148 | 0.75 | 0.65 | 0.85 | 0 |
| 43315 | 136 | 0.563 | 0.279 | 0.6 | 0.4 | 0.75 | 2.21 |
| 43514 | 129 | 0.555 | 0.24 | 0.6 | 0.45 | 0.75 | 2.33 |
| 43542 | 138 | 0.479 | 0.206 | 0.45 | 0.3 | 0.65 | 1.45 |
| 43555 | 148 | 0.116 | 0.397 | 0.2 | 0.05 | 0.35 | 21.62 |
| 44125 | 112 | 0.53 | 0.297 | 0.6 | 0.4 | 0.725 | 2.68 |
| 44345 | 124 | 0.309 | 0.372 | 0.35 | 0.1 | 0.575 | 11.29 |
| 44553 | 132 | 0.18 | 0.403 | 0.25 | 0.05 | 0.4 | 19.7 |
| 45133 | 142 | 0.531 | 0.285 | 0.575 | 0.4 | 0.75 | 2.82 |
| 45144 | 139 | 0.413 | 0.308 | 0.45 | 0.3 | 0.6 | 6.47 |
| 45233 | 130 | 0.537 | 0.207 | 0.55 | 0.4 | 0.65 | 0.77 |
| 45413 | 137 | 0.521 | 0.276 | 0.55 | 0.4 | 0.7 | 2.92 |
| 51152 | 138 | 0.255 | 0.418 | 0.35 | 0.15 | 0.5 | 16.67 |
| 51451 | 143 | 0.248 | 0.382 | 0.3 | 0.15 | 0.45 | 16.08 |
| 52215 | 138 | 0.48 | 0.317 | 0.5 | 0.35 | 0.7 | 3.62 |
| 52335 | 148 | 0.368 | 0.359 | 0.425 | 0.3 | 0.6 | 8.78 |
| 52431 | 141 | 0.512 | 0.292 | 0.55 | 0.35 | 0.7 | 3.55 |
| 52455 | 133 | 0.027 | 0.398 | 0.15 | -0.3 | 0.3 | 33.83 |
| 53221 | 116 | 0.542 | 0.291 | 0.55 | 0.45 | 0.75 | 5.17 |
| 53243 | 139 | 0.451 | 0.262 | 0.45 | 0.35 | 0.65 | 2.88 |
| 53244 | 142 | 0.432 | 0.245 | 0.45 | 0.3 | 0.6 | 2.82 |
| 53412 | 133 | 0.517 | 0.298 | 0.55 | 0.4 | 0.7 | 4.51 |
| 54153 | 139 | 0.25 | 0.392 | 0.35 | 0.15 | 0.5 | 16.55 |
| 54231 | 142 | 0.514 | 0.265 | 0.5 | 0.4 | 0.7 | 2.11 |
| 54342 | 122 | 0.35 | 0.346 | 0.4 | 0.25 | 0.55 | 9.02 |
| 55225 | 135 | 0.171 | 0.439 | 0.3 | 0.05 | 0.45 | 22.96 |
| 55233 | 128 | 0.316 | 0.381 | 0.35 | 0.2 | 0.55 | 10.94 |
| 55424 | 127 | 0.213 | 0.408 | 0.3 | 0.1 | 0.45 | 15.75 |
| 55555 | 1473 | -0.483 | 0.406 | -0.5 | -0.85 | -0.15 | 78.75 |

*SD*, standard deviation

Annex 5 Composite time trade-off (cTTO), discrete choice experiment (DCE) and hybrid models tested

| Model | Consistency of parameters achieved | Number of non-significant parameters | Non-Significant parameters |
| --- | --- | --- | --- |
| OLS | Yes | 2 | MO2; MO3 |
| ROLS | No | 2 | MO2; MO3 |
| RME | No | 2 | MO2 |
| IME | Yes | 3 | MO2; MO3; constant |
| TOB | No | 2 | MO2; MO3 |
| TOBRME | No | 2 | MO2; MO3 |
| TOBIME | No | 2 | MO2; MO3 |
| IRM | Yes | 0 | 0 |
| IRMC | Yes | 0 | 0 |
| IRMCRME | No | 2 | M02; MO3 |
| IRMCIME | No | 3 | MO2; MO3; constant |
| RCMRME | No | 2 | MO2; MO3 |
| RCMIME | No | 2 | MO2; MO3 |
| CLOGIT | No | 0 | N/A |
| PROBIT | Yes | 1 | AD2 |
| HPROBIT | Yes | 1 | AD2 |
| PROBITRME | Yes | 1 | AD2 |
| PROBITIME | Yes | 2 | AD2 |
| SHMC | Yes | 0 | N/A |
| CHMC | Yes | 0 | N/A |
| HMH | Yes | 0 | N/A |
| HMHC | Yes | 0 | N/A |

OLS, ordinary least-squares; ROLS, robust ordinary least-squares; RME, respondent-level mixed effects; IME, interviewer-level mixed effects; TOB, tobit; TOBRME, tobit with respondent-level mixed effects; IRM, interval regression model; IRMC, interval regression model censored at -1; IRMCRME, interval regression model censored at -1 with respondent-level mixed effects; IRMCIME, interval regression model censored at -1 with interviewer-level mixed effects; RCMRME, random coefficient model with respondent-level mixed effects; RCMIME, random coefficient model with interviewer-level mixed effects; SHMC, simple hybrid model with constant; CHMC, censored hybrid model with constant; PROBIT, probit model; HPROBIT, heteroskedastic probit model; PROBITRME, probit model with respondent-level mixed effects; PROBITIME, probit model with interviewer-level mixed effects; HMH, hybrid model heteroskedastic without constant; HMHC, censored hybrid model heteroskedastic without constant;

Annex 6 Full value set model for the Romanian version of EQ-5D-5L

| **Independent variables of the model** | **Coefficient** | **SE** | **p-value** | **95% CI** | |
| --- | --- | --- | --- | --- | --- |
| MO2 | 0.039 | 0.004 | 0.000 | 0.032 | 0.046 |
| MO3 | 0.056 | 0.005 | 0.000 | 0.046 | 0.065 |
| MO4 | 0.107 | 0.005 | 0.000 | 0.097 | 0.117 |
| MO5 | 0.293 | 0.005 | 0.000 | 0.283 | 0.304 |
| SC2 | 0.048 | 0.003 | 0.000 | 0.041 | 0.054 |
| SC3 | 0.052 | 0.005 | 0.000 | 0.043 | 0.061 |
| SC4 | 0.098 | 0.005 | 0.000 | 0.088 | 0.107 |
| SC5 | 0.233 | 0.005 | 0.000 | 0.223 | 0.242 |
| UA2 | 0.039 | 0.003 | 0.000 | 0.033 | 0.046 |
| UA3 | 0.058 | 0.005 | 0.000 | 0.049 | 0.067 |
| UA4 | 0.111 | 0.005 | 0.000 | 0.102 | 0.12 |
| UA5 | 0.203 | 0.005 | 0.000 | 0.194 | 0.212 |
| PD2 | 0.053 | 0.003 | 0.000 | 0.047 | 0.059 |
| PD3 | 0.077 | 0.005 | 0.000 | 0.068 | 0.087 |
| PD4 | 0.156 | 0.005 | 0.000 | 0.147 | 0.165 |
| PD5 | 0.375 | 0.006 | 0.000 | 0.363 | 0.387 |
| AD2 | 0.038 | 0.003 | 0.000 | 0.032 | 0.044 |
| AD3 | 0.059 | 0.005 | 0.000 | 0.05 | 0.068 |
| AD4 | 0.11 | 0.004 | 0.000 | 0.102 | 0.119 |
| AD5 | 0.218 | 0.005 | 0.000 | 0.209 | 0.228 |
| **Model for lnsigma** |  |  |  |  |  |
| MO2 | 0.216 | 0.021 | 0.000 | 0.174 | 0.257 |
| MO3 | 0.323 | 0.022 | 0.000 | 0.28 | 0.366 |
| MO4 | 0.354 | 0.024 | 0.000 | 0.306 | 0.402 |
| MO5 | 0.512 | 0.02 | 0.000 | 0.473 | 0.551 |
| SC2 | 0.17 | 0.021 | 0.000 | 0.129 | 0.211 |
| SC3 | 0.176 | 0.024 | 0.000 | 0.13 | 0.222 |
| SC4 | 0.279 | 0.022 | 0.000 | 0.235 | 0.322 |
| SC5 | 0.464 | 0.02 | 0.000 | 0.425 | 0.503 |
| UA2 | 0.118 | 0.022 | 0.000 | 0.075 | 0.162 |
| UA3 | 0.288 | 0.023 | 0.000 | 0.243 | 0.334 |
| UA4 | 0.312 | 0.023 | 0.000 | 0.267 | 0.357 |
| UA5 | 0.162 | 0.021 | 0.000 | 0.121 | 0.203 |
| PD2 | 0.139 | 0.02 | 0.000 | 0.099 | 0.178 |
| PD3 | 0.106 | 0.023 | 0.000 | 0.06 | 0.151 |
| PD4 | 0.188 | 0.02 | 0.000 | 0.149 | 0.227 |
| PD5 | 0.566 | 0.022 | 0.000 | 0.523 | 0.608 |
| AD2 | 0.08 | 0.021 | 0.000 | 0.038 | 0.122 |
| AD3 | 0.157 | 0.024 | 0.000 | 0.11 | 0.203 |
| AD4 | 0.244 | 0.023 | 0.000 | 0.2 | 0.289 |
| AD5 | 0.333 | 0.02 | 0.000 | 0.293 | 0.373 |

MO, mobility; SC, self-care; UA, usual activities; PD, pain discomfort; AD, anxiety depression; SE, standard error; CI, confidence interval

Annex 7 Sociodemographic characteristics of the sample corresponding to the V1 dataset

| **Variable** | **Category** | **V1 (n=1649)** | | **Weighted V1 (n=1649)** | | **General population** |
| --- | --- | --- | --- | --- | --- | --- |
|  |  | **n** | **%** | **n** | **%** | **%** |
| Gender | Female | 1072 | 65.0 | 857 | 52.0 | 52.0 |
| Residence area | Urban | 1212 | 73.5 | 895 | 54.2 | 55.2 |
| Education level | No formal education | 7 | 0.4 | 13 | 0.8 | 2.0 |
|  | Low | 192 | 11.6 | 255 | 15.5 | 36.9 |
|  | Medium | 830 | 50.3 | 877 | 53.2 | 45.2 |
|  | High | 611 | 37.1 | 494 | 30.0 | 15.9 |
|  | No response | 9 | 0.5 | 9 | 0.6 |  |
| Occupation | Employed | 973 | 59.0 | 867 | 52.5 | 52.1 |
|  | Unemployed | 35 | 2.1 | 54 | 3.3 | 3.9 |
|  | Retired | 415 | 25.2 | 442 | 26.8 | 26.2 |
|  | Stay at home/domestic | 117 | 7.1 | 147 | 8.9 | 7.1 |
|  | In education | 92 | 5.6 | 111 | 6.7 | 4.8 |
|  | No response | 17 | 1.0 | 28 | 1.7 |  |
| Income | Below the average | 705 | 42.8 | 773 | 46.9 | 41.4 |
|  | Average | 298 | 18.1 | 278 | 16.8 | 30.7 |
|  | Above the average | 513 | 31.1 | 447 | 27.1 | 27.9 |
|  | No response | 133 | 8.1 | 152 | 9.2 |  |
| Experience with serious illness | In self | 327 | 19.8 | 347 | 21.0 |  |
|  | In family | 763 | 46.3 | 740 | 44.9 | N/A |
|  | In caring for others | 270 | 16.4 | 240 | 14.6 |  |
| Self-rated health using EQ-5D-5L | 11111 | 871 | 52.8 | 829 | 50.3 | N/A |
|  | Any other health state | 778 | 47.2 | 820 | 49.7 |  |

V1 dataset includes all interviews performed with the exception of those interviews performed by interviewers that were subsequently excluded from the interviewers’ team due to quality control issues.

Annex 8 Full Romanian EQ-5D-5L model run on dataset V1

| **Independent variables of the model** | **Coefficient** | **SE** | **p-value** | **95% CI** | |
| --- | --- | --- | --- | --- | --- |
| MO2 | 0.037 | 0.003 | 0.000 | 0.031 | 0.044 |
| MO3 | 0.055 | 0.005 | 0.000 | 0.046 | 0.065 |
| MO4 | 0.105 | 0.005 | 0.000 | 0.096 | 0.114 |
| MO5 | 0.29 | 0.005 | 0.000 | 0.281 | 0.300 |
| SC2 | 0.048 | 0.003 | 0.000 | 0.042 | 0.054 |
| SC3 | 0.051 | 0.004 | 0.000 | 0.043 | 0.060 |
| SC4 | 0.101 | 0.005 | 0.000 | 0.092 | 0.110 |
| SC5 | 0.231 | 0.005 | 0.000 | 0.222 | 0.240 |
| UA2 | 0.039 | 0.003 | 0.000 | 0.032 | 0.045 |
| UA3 | 0.059 | 0.004 | 0.000 | 0.051 | 0.068 |
| UA4 | 0.113 | 0.005 | 0.000 | 0.104 | 0.122 |
| UA5 | 0.203 | 0.004 | 0.000 | 0.194 | 0.211 |
| PD2 | 0.054 | 0.003 | 0.000 | 0.049 | 0.060 |
| PD3 | 0.077 | 0.005 | 0.000 | 0.068 | 0.086 |
| PD4 | 0.153 | 0.004 | 0.000 | 0.145 | 0.162 |
| PD5 | 0.361 | 0.006 | 0.000 | 0.35 | 0.373 |
| AD2 | 0.036 | 0.003 | 0.000 | 0.031 | 0.042 |
| AD3 | 0.056 | 0.005 | 0.000 | 0.047 | 0.065 |
| AD4 | 0.108 | 0.004 | 0.000 | 0.1 | 0.116 |
| AD5 | 0.214 | 0.004 | 0.000 | 0.205 | 0.222 |
| **Model for lnsigma** |  |  |  |  |  |
| MO2 | 0.22 | 0.02 | 0.000 | 0.181 | 0.259 |
| MO3 | 0.327 | 0.021 | 0.000 | 0.286 | 0.368 |
| MO4 | 0.342 | 0.023 | 0.000 | 0.296 | 0.387 |
| MO5 | 0.518 | 0.019 | 0.000 | 0.48 | 0.555 |
| SC2 | 0.149 | 0.02 | 0.000 | 0.11 | 0.187 |
| SC3 | 0.168 | 0.022 | 0.000 | 0.124 | 0.212 |
| SC4 | 0.271 | 0.021 | 0.000 | 0.23 | 0.312 |
| SC5 | 0.439 | 0.019 | 0.000 | 0.402 | 0.477 |
| UA2 | 0.107 | 0.021 | 0.000 | 0.066 | 0.149 |
| UA3 | 0.273 | 0.022 | 0.000 | 0.23 | 0.316 |
| UA4 | 0.315 | 0.022 | 0.000 | 0.273 | 0.358 |
| UA5 | 0.173 | 0.02 | 0.000 | 0.135 | 0.212 |
| PD2 | 0.129 | 0.019 | 0.000 | 0.092 | 0.166 |
| PD3 | 0.102 | 0.022 | 0.000 | 0.059 | 0.145 |
| PD4 | 0.188 | 0.019 | 0.000 | 0.151 | 0.225 |
| PD5 | 0.553 | 0.02 | 0.000 | 0.513 | 0.593 |
| AD2 | 0.067 | 0.02 | 0.001 | 0.027 | 0.107 |
| AD3 | 0.163 | 0.022 | 0.000 | 0.119 | 0.207 |
| AD4 | 0.243 | 0.022 | 0.000 | 0.2 | 0.285 |
| AD5 | 0.33 | 0.019 | 0.000 | 0.292 | 0.368 |

V1 dataset includes all interviews performed with the exception of those interviews performed by interviewers that were subsequently excluded from the interviewers’ team due to quality control issues; SE, standard error; CI, confidence interval; MO, mobility; SC, self-care; UA, usual activities; PD, pain discomfort; AD, anxiety depression.

Annex 9 Full Romanian EQ-5D-5L model run on weighted V6 sample

| **Independent variables of the model** | **Coefficient** | **SE** | **p-value** | **95% CI** | |
| --- | --- | --- | --- | --- | --- |
| MO2 | 0.04 | 0.004 | 0.000 | 0.033 | 0.047 |
| MO3 | 0.055 | 0.005 | 0.000 | 0.046 | 0.065 |
| MO4 | 0.107 | 0.005 | 0.000 | 0.097 | 0.116 |
| MO5 | 0.296 | 0.005 | 0.000 | 0.286 | 0.306 |
| **SC2** | **0.055** | **0.003** | **0.000** | **0.048** | **0.061** |
| **SC3** | **0.052** | **0.005** | **0.000** | **0.043** | **0.061** |
| SC4 | 0.096 | 0.005 | 0.000 | 0.087 | 0.106 |
| SC5 | 0.241 | 0.005 | 0.000 | 0.232 | 0.251 |
| UA2 | 0.045 | 0.003 | 0.000 | 0.038 | 0.051 |
| UA3 | 0.06 | 0.005 | 0.000 | 0.051 | 0.069 |
| UA4 | 0.123 | 0.005 | 0.000 | 0.113 | 0.132 |
| UA5 | 0.22 | 0.004 | 0.000 | 0.211 | 0.229 |
| PD2 | 0.053 | 0.003 | 0.000 | 0.047 | 0.059 |
| PD3 | 0.075 | 0.005 | 0.000 | 0.066 | 0.084 |
| PD4 | 0.142 | 0.005 | 0.000 | 0.133 | 0.151 |
| PD5 | 0.359 | 0.006 | 0.000 | 0.348 | 0.371 |
| AD2 | 0.038 | 0.003 | 0.000 | 0.033 | 0.044 |
| AD3 | 0.058 | 0.005 | 0.000 | 0.049 | 0.067 |
| AD4 | 0.105 | 0.004 | 0.000 | 0.096 | 0.113 |
| AD5 | 0.22 | 0.005 | 0.000 | 0.211 | 0.229 |
| **Model for lnsigma** |  |  |  |  |  |
| MO2 | 0.218 | 0.021 | 0.000 | 0.176 | 0.260 |
| MO3 | 0.333 | 0.022 | 0.000 | 0.29 | 0.377 |
| MO4 | 0.364 | 0.025 | 0.000 | 0.316 | 0.412 |
| MO5 | 0.495 | 0.02 | 0.000 | 0.456 | 0.534 |
| SC2 | 0.23 | 0.021 | 0.000 | 0.19 | 0.271 |
| SC3 | 0.194 | 0.024 | 0.000 | 0.148 | 0.240 |
| SC4 | 0.287 | 0.022 | 0.000 | 0.244 | 0.330 |
| SC5 | 0.5 | 0.02 | 0.000 | 0.461 | 0.539 |
| UA2 | 0.185 | 0.022 | 0.000 | 0.141 | 0.229 |
| UA3 | 0.329 | 0.024 | 0.000 | 0.283 | 0.376 |
| UA4 | 0.357 | 0.023 | 0.000 | 0.312 | 0.402 |
| UA5 | 0.173 | 0.021 | 0.000 | 0.132 | 0.214 |
| PD2 | 0.171 | 0.02 | 0.000 | 0.131 | 0.210 |
| PD3 | 0.112 | 0.023 | 0.000 | 0.066 | 0.157 |
| PD4 | 0.164 | 0.02 | 0.000 | 0.124 | 0.203 |
| PD5 | 0.598 | 0.022 | 0.000 | 0.555 | 0.641 |
| AD2 | 0.155 | 0.022 | 0.000 | 0.113 | 0.198 |
| AD3 | 0.208 | 0.024 | 0.000 | 0.161 | 0.255 |
| AD4 | 0.267 | 0.023 | 0.000 | 0.222 | 0.311 |
| AD5 | 0.351 | 0.02 | 0.000 | 0.311 | 0.391 |

MO, mobility; SC, self-care; UA, usual activities; PD, pain discomfort; AD, anxiety depression; SE, standard error; CI, confidence interval; **values in bold**, inconsistent parameters

V6, the dataset that includes those interviews that were valid after all exclusion criteria have been applied

Annex 10 Observed and predicted values for the 86 EQ-5D-5L health states included in the composite time trade-off (cTTO) tasks

HMHC, censored hybrid model heteroskedastic without constant

Annex 11 Comparison between observed cTTO values for the 86 EQ-5D-5L health states common to the Hungarian, Romanian and Polish EQ-5D-5L valuation studies

| **Health** | **cTTO Poland** | | **cTTO Hungary** | | **cTTO Romania** | | | **Difference Romania vs Hungary** | | | **Difference Romania vs Poland** | | |
| --- | --- | --- | --- | --- | --- | --- | --- | --- | --- | --- | --- | --- | --- |
| **state** | **Mean** | **SD** | **Mean** | **SD** | **n** | **Mean** | **SD** | **Diff.** | **z** | **p-value** | **Diff.** | **z** | **p-value** |
| 11112 | 0.981 | 0.026 | 0.969 | 0.073 | 302 | 0.958 | 0.053 | 0.011 | -3.61 | 0.0002 | 0.023 | -7.54 | 0.0000 |
| 11121 | 0.968 | 0.046 | 0.957 | 0.113 | 279 | 0.949 | 0.083 | 0.008 | -1.61 | 0.0537 | 0.019 | -3.82 | 0.0001 |
| 11122 | 0.949 | 0.064 | 0.923 | 0.09 | 144 | 0.926 | 0.074 | -0.003 | 0.49 | 0.3133 | 0.023 | -3.73 | 0.0001 |
| 11211 | 0.973 | 0.038 | 0.964 | 0.069 | 295 | 0.946 | 0.069 | 0.018 | -4.48 | 0.0000 | 0.027 | -6.72 | 0.0000 |
| 11212 | 0.959 | 0.042 | 0.92 | 0.125 | 134 | 0.899 | 0.076 | 0.021 | -3.20 | 0.0007 | 0.060 | -9.14 | 0.0000 |
| 11221 | 0.94 | 0.103 | 0.92 | 0.119 | 142 | 0.903 | 0.078 | 0.017 | -2.60 | 0.0047 | 0.037 | -5.65 | 0.0000 |
| 11235 | 0.725 | 0.209 | 0.564 | 0.32 | 142 | 0.688 | 0.181 | -0.124 | 8.16 | 0.0000 | 0.037 | -2.44 | 0.0074 |
| 11414 | 0.784 | 0.246 | 0.541 | 0.306 | 137 | 0.734 | 0.177 | -0.193 | 12.76 | 0.0000 | 0.050 | -3.31 | 0.0005 |
| 11421 | 0.831 | 0.275 | 0.711 | 0.205 | 133 | 0.786 | 0.146 | -0.075 | 5.92 | 0.0000 | 0.045 | -3.55 | 0.0002 |
| 11425 | 0.649 | 0.377 | 0.427 | 0.361 | 139 | 0.614 | 0.257 | -0.187 | 8.58 | 0.0000 | 0.035 | -1.61 | 0.0542 |
| 12111 | 0.965 | 0.044 | 0.951 | 0.108 | 300 | 0.950 | 0.058 | 0.001 | -0.30 | 0.3826 | 0.015 | -4.48 | 0.0000 |
| 12112 | 0.945 | 0.099 | 0.909 | 0.142 | 132 | 0.885 | 0.165 | 0.024 | -1.67 | 0.0473 | 0.060 | -4.18 | 0.0000 |
| 12121 | 0.95 | 0.052 | 0.924 | 0.083 | 138 | 0.891 | 0.099 | 0.033 | -3.92 | 0.0000 | 0.059 | -7.00 | 0.0000 |
| 12244 | 0.57 | 0.39 | 0.398 | 0.344 | 129 | 0.631 | 0.220 | -0.233 | 12.03 | 0.0000 | -0.061 | 3.15 | 0.0008 |
| 12334 | 0.777 | 0.183 | 0.571 | 0.28 | 142 | 0.720 | 0.146 | -0.149 | 12.16 | 0.0000 | 0.057 | -4.65 | 0.0000 |
| 12344 | 0.504 | 0.46 | 0.284 | 0.401 | 121 | 0.624 | 0.186 | -0.340 | 20.11 | 0.0000 | -0.120 | 7.10 | 0.0000 |
| 12513 | 0.721 | 0.31 | 0.596 | 0.263 | 125 | 0.680 | 0.165 | -0.084 | 5.69 | 0.0000 | 0.041 | -2.78 | 0.0027 |
| 12514 | 0.673 | 0.315 | 0.43 | 0.383 | 143 | 0.650 | 0.215 | -0.220 | 12.24 | 0.0000 | 0.023 | -1.28 | 0.1004 |
| 12543 | 0.517 | 0.412 | 0.294 | 0.447 | 137 | 0.568 | 0.205 | -0.274 | 15.64 | 0.0000 | -0.051 | 2.91 | 0.0018 |
| 13122 | 0.922 | 0.077 | 0.806 | 0.149 | 149 | 0.831 | 0.105 | -0.025 | 2.91 | 0.0018 | 0.091 | -10.58 | 0.0000 |
| 13224 | 0.749 | 0.272 | 0.57 | 0.32 | 138 | 0.728 | 0.144 | -0.158 | 12.89 | 0.0000 | 0.021 | -1.71 | 0.0433 |
| 13313 | 0.878 | 0.162 | 0.708 | 0.218 | 129 | 0.789 | 0.127 | -0.081 | 7.24 | 0.0000 | 0.089 | -7.96 | 0.0000 |
| 14113 | 0.881 | 0.11 | 0.681 | 0.255 | 145 | 0.805 | 0.104 | -0.124 | 14.36 | 0.0000 | 0.076 | -8.80 | 0.0000 |
| 14554 | 0.143 | 0.55 | -0.194 | 0.516 | 125 | 0.244 | 0.374 | -0.438 | 13.09 | 0.0000 | -0.101 | 3.02 | 0.0013 |
| 15151 | 0.303 | 0.506 | 0.258 | 0.457 | 144 | 0.374 | 0.396 | -0.116 | 3.52 | 0.0002 | -0.071 | 2.15 | 0.0157 |
| 21111 | 0.969 | 0.055 | 0.971 | 0.083 | 274 | 0.948 | 0.096 | 0.023 | -3.97 | 0.0000 | 0.021 | -3.62 | 0.0001 |
| 21112 | 0.94 | 0.136 | 0.91 | 0.157 | 126 | 0.906 | 0.093 | 0.004 | -0.48 | 0.3146 | 0.034 | -4.10 | 0.0000 |
| 21315 | 0.746 | 0.314 | 0.541 | 0.322 | 146 | 0.664 | 0.256 | -0.123 | 5.81 | 0.0000 | 0.082 | -3.87 | 0.0001 |
| 21334 | 0.786 | 0.163 | 0.541 | 0.331 | 137 | 0.697 | 0.157 | -0.156 | 11.63 | 0.0000 | 0.089 | -6.64 | 0.0000 |
| 21345 | 0.512 | 0.371 | 0.313 | 0.385 | 130 | 0.527 | 0.281 | -0.214 | 8.68 | 0.0000 | -0.015 | 0.61 | 0.2714 |
| 21444 | 0.53 | 0.379 | 0.065 | 0.466 | 128 | 0.564 | 0.226 | -0.499 | 24.98 | 0.0000 | -0.034 | 1.70 | 0.0444 |
| 22434 | 0.714 | 0.286 | 0.395 | 0.364 | 132 | 0.670 | 0.159 | -0.275 | 19.87 | 0.0000 | 0.044 | -3.18 | 0.0007 |
| 23152 | 0.408 | 0.467 | 0.446 | 0.373 | 129 | 0.492 | 0.267 | -0.046 | 1.96 | 0.0252 | -0.084 | 3.57 | 0.0002 |
| 23242 | 0.563 | 0.392 | 0.503 | 0.325 | 141 | 0.687 | 0.193 | -0.184 | 11.32 | 0.0000 | -0.124 | 7.63 | 0.0000 |
| 23514 | 0.634 | 0.373 | 0.374 | 0.44 | 132 | 0.645 | 0.175 | -0.271 | 17.79 | 0.0000 | -0.011 | 0.72 | 0.2351 |
| 24342 | 0.569 | 0.364 | 0.347 | 0.4 | 133 | 0.588 | 0.251 | -0.241 | 11.07 | 0.0000 | -0.019 | 0.87 | 0.1913 |
| 24443 | 0.507 | 0.42 | 0.122 | 0.481 | 132 | 0.547 | 0.205 | -0.425 | 23.82 | 0.0000 | -0.040 | 2.24 | 0.0125 |
| 24445 | 0.263 | 0.496 | -0.134 | 0.493 | 149 | 0.444 | 0.258 | -0.578 | 27.35 | 0.0000 | -0.181 | 8.56 | 0.0000 |
| 24553 | 0.19 | 0.569 | -0.083 | 0.506 | 136 | 0.294 | 0.368 | -0.377 | 11.95 | 0.0000 | -0.104 | 3.30 | 0.0005 |
| 25122 | 0.637 | 0.43 | 0.49 | 0.418 | 132 | 0.691 | 0.170 | -0.201 | 13.58 | 0.0000 | -0.054 | 3.65 | 0.0001 |
| 25222 | 0.693 | 0.341 | 0.52 | 0.428 | 140 | 0.652 | 0.291 | -0.132 | 5.37 | 0.0000 | 0.041 | -1.67 | 0.0477 |
| 25331 | 0.662 | 0.363 | 0.422 | 0.375 | 131 | 0.610 | 0.311 | -0.188 | 6.92 | 0.0000 | 0.052 | -1.91 | 0.0278 |
| 31514 | 0.671 | 0.301 | 0.376 | 0.339 | 139 | 0.640 | 0.181 | -0.264 | 17.20 | 0.0000 | 0.031 | -2.02 | 0.0217 |
| 31524 | 0.68 | 0.338 | 0.361 | 0.393 | 131 | 0.603 | 0.229 | -0.242 | 12.10 | 0.0000 | 0.077 | -3.85 | 0.0001 |
| 31525 | 0.547 | 0.391 | 0.243 | 0.411 | 130 | 0.531 | 0.263 | -0.288 | 12.49 | 0.0000 | 0.016 | -0.69 | 0.2440 |
| 32314 | 0.787 | 0.267 | 0.55 | 0.339 | 136 | 0.714 | 0.160 | -0.164 | 11.95 | 0.0000 | 0.073 | -5.32 | 0.0000 |
| 32443 | 0.601 | 0.285 | 0.226 | 0.455 | 138 | 0.576 | 0.183 | -0.350 | 22.47 | 0.0000 | 0.025 | -1.60 | 0.0543 |
| 33253 | 0.315 | 0.512 | 0.335 | 0.409 | 141 | 0.401 | 0.319 | -0.066 | 2.46 | 0.0070 | -0.086 | 3.20 | 0.0007 |
| 34155 | 0.112 | 0.567 | -0.112 | 0.532 | 145 | 0.324 | 0.324 | -0.436 | 16.20 | 0.0000 | -0.212 | 7.88 | 0.0000 |
| 34232 | 0.736 | 0.256 | 0.49 | 0.344 | 141 | 0.678 | 0.173 | -0.188 | 12.90 | 0.0000 | 0.058 | -3.98 | 0.0000 |
| 34244 | 0.5 | 0.424 | 0.046 | 0.525 | 131 | 0.552 | 0.229 | -0.506 | 25.29 | 0.0000 | -0.052 | 2.60 | 0.0047 |
| 34515 | 0.505 | 0.379 | 0.081 | 0.524 | 140 | 0.453 | 0.301 | -0.372 | 14.62 | 0.0000 | 0.052 | -2.04 | 0.0205 |
| 35143 | 0.483 | 0.43 | 0.177 | 0.434 | 134 | 0.507 | 0.322 | -0.330 | 11.86 | 0.0000 | -0.024 | 0.86 | 0.1941 |
| 35245 | 0.339 | 0.469 | -0.038 | 0.507 | 142 | 0.362 | 0.355 | -0.400 | 13.43 | 0.0000 | -0.023 | 0.77 | 0.2200 |
| 35311 | 0.635 | 0.464 | 0.461 | 0.378 | 142 | 0.632 | 0.321 | -0.171 | 6.35 | 0.0000 | 0.003 | -0.11 | 0.4557 |
| 35332 | 0.627 | 0.417 | 0.304 | 0.436 | 138 | 0.578 | 0.261 | -0.274 | 12.33 | 0.0000 | 0.049 | -2.21 | 0.0137 |
| 42115 | 0.632 | 0.361 | 0.312 | 0.414 | 138 | 0.617 | 0.238 | -0.305 | 15.05 | 0.0000 | 0.015 | -0.74 | 0.2295 |
| 42321 | 0.757 | 0.322 | 0.578 | 0.233 | 136 | 0.725 | 0.148 | -0.147 | 11.58 | 0.0000 | 0.032 | -2.52 | 0.0058 |
| 43315 | 0.661 | 0.336 | 0.199 | 0.487 | 136 | 0.563 | 0.279 | -0.364 | 15.21 | 0.0000 | 0.098 | -4.10 | 0.0000 |
| 43514 | 0.596 | 0.309 | 0.092 | 0.485 | 129 | 0.555 | 0.240 | -0.463 | 21.91 | 0.0000 | 0.041 | -1.94 | 0.0262 |
| 43542 | 0.416 | 0.494 | 0.124 | 0.491 | 138 | 0.479 | 0.206 | -0.355 | 20.24 | 0.0000 | -0.063 | 3.59 | 0.0002 |
| 43555 | 0.003 | 0.552 | -0.297 | 0.56 | 148 | 0.116 | 0.397 | -0.413 | 12.66 | 0.0000 | -0.113 | 3.46 | 0.0003 |
| 44125 | 0.458 | 0.443 | 0.113 | 0.449 | 112 | 0.530 | 0.297 | -0.417 | 14.86 | 0.0000 | -0.072 | 2.57 | 0.0052 |
| 44345 | 0.25 | 0.523 | -0.228 | 0.508 | 124 | 0.309 | 0.372 | -0.537 | 16.07 | 0.0000 | -0.059 | 1.77 | 0.0387 |
| 44553 | 0.089 | 0.547 | -0.339 | 0.509 | 132 | 0.180 | 0.403 | -0.519 | 14.80 | 0.0000 | -0.091 | 2.59 | 0.0047 |
| 45133 | 0.533 | 0.405 | 0.3 | 0.446 | 142 | 0.531 | 0.285 | -0.231 | 9.66 | 0.0000 | 0.002 | -0.08 | 0.4667 |
| 45144 | 0.31 | 0.512 | -0.177 | 0.546 | 139 | 0.413 | 0.308 | -0.590 | 22.58 | 0.0000 | -0.103 | 3.94 | 0.0000 |
| 45233 | 0.497 | 0.485 | 0.153 | 0.467 | 130 | 0.537 | 0.207 | -0.384 | 21.15 | 0.0000 | -0.040 | 2.20 | 0.0138 |
| 45413 | 0.552 | 0.415 | 0.08 | 0.48 | 137 | 0.521 | 0.276 | -0.441 | 18.70 | 0.0000 | 0.031 | -1.31 | 0.0943 |
| 51152 | 0.232 | 0.553 | 0.03 | 0.518 | 138 | 0.255 | 0.418 | -0.225 | 6.32 | 0.0000 | -0.023 | 0.65 | 0.2590 |
| 51451 | 0.202 | 0.531 | 0.003 | 0.471 | 143 | 0.248 | 0.382 | -0.245 | 7.67 | 0.0000 | -0.046 | 1.44 | 0.0749 |
| 52215 | 0.38 | 0.56 | 0.143 | 0.556 | 138 | 0.480 | 0.317 | -0.337 | 12.49 | 0.0000 | -0.100 | 3.71 | 0.0001 |
| 52335 | 0.404 | 0.514 | -0.062 | 0.538 | 148 | 0.368 | 0.359 | -0.430 | 14.57 | 0.0000 | 0.036 | -1.22 | 0.1112 |
| 52431 | 0.62 | 0.362 | 0.234 | 0.46 | 141 | 0.512 | 0.292 | -0.278 | 11.31 | 0.0000 | 0.108 | -4.39 | 0.0000 |
| 52455 | -0.004 | 0.564 | -0.34 | 0.488 | 133 | 0.027 | 0.398 | -0.367 | 10.63 | 0.0000 | -0.031 | 0.90 | 0.1845 |
| 53221 | 0.666 | 0.392 | 0.391 | 0.457 | 116 | 0.542 | 0.291 | -0.151 | 5.59 | 0.0000 | 0.124 | -4.59 | 0.0000 |
| 53243 | 0.44 | 0.49 | 0.003 | 0.471 | 139 | 0.451 | 0.262 | -0.448 | 20.16 | 0.0000 | -0.011 | 0.49 | 0.3103 |
| 53244 | 0.362 | 0.529 | -0.124 | 0.501 | 142 | 0.432 | 0.245 | -0.556 | 27.04 | 0.0000 | -0.070 | 3.40 | 0.0003 |
| 53412 | 0.509 | 0.537 | 0.28 | 0.443 | 133 | 0.517 | 0.298 | -0.237 | 9.17 | 0.0000 | -0.008 | 0.31 | 0.3784 |
| 54153 | 0.209 | 0.55 | -0.132 | 0.542 | 139 | 0.250 | 0.392 | -0.382 | 11.49 | 0.0000 | -0.041 | 1.23 | 0.1088 |
| 54231 | 0.579 | 0.451 | 0.201 | 0.471 | 142 | 0.514 | 0.265 | -0.313 | 14.07 | 0.0000 | 0.065 | -2.92 | 0.0017 |
| 54342 | 0.302 | 0.53 | -0.04 | 0.464 | 122 | 0.350 | 0.346 | -0.390 | 12.45 | 0.0000 | -0.048 | 1.53 | 0.0627 |
| 55225 | 0.156 | 0.576 | -0.179 | 0.533 | 135 | 0.171 | 0.439 | -0.350 | 9.26 | 0.0000 | -0.015 | 0.40 | 0.3457 |
| 55233 | 0.293 | 0.59 | -0.067 | 0.522 | 128 | 0.316 | 0.381 | -0.383 | 11.37 | 0.0000 | -0.023 | 0.68 | 0.2473 |
| 55424 | 0.211 | 0.579 | -0.372 | 0.493 | 127 | 0.213 | 0.408 | -0.585 | 16.16 | 0.0000 | -0.002 | 0.06 | 0.4780 |
| 55555 | -0.433 | 0.56 | -0.642 | 0.465 | 1473 | -0.483 | 0.406 | -0.159 | 15.03 | 0.0000 | 0.050 | -4.73 | 0.0000 |

*cTTO*, composite time trade-off

Annex 12 Comparison between the Romanian EQ-5D-5L value set and the Romanian EQ-5D-3L value set

| **Comparison Criteria** | **RO EQ-5D-5L value set** | **RO EQ-5D-3L value set** |
| --- | --- | --- |
| **Valuation method** | Hybrid (TTO/DCE) | TTO |
| **Dimension ordering (most to least important)** | PD-MO-SC-AD-UA | MO-PD-SC-AD-UA |
| **Number of health states** | 3125 | 243 |
| **Maximum value (health state)** | 1.000 (11111) | 1.000 (11111) |
| **Second highest value (health state)** | 0.962 (11112) | 0.93 (21111) |
| **Values for mildest health states** |  |  |
| 21111 | 0.961 | 0.93 |
| 12111 | 0.952 | 0.927 |
| 11211 | 0.961 | 0.923 |
| 11121 | 0.947 | 0.896 |
| 11112 | 0.962 | 0.914 |
| **Values for most severe EQ-5D-3L health states** | | |
| 23333 | * | -0.042 |
| 32333 | * | -0.233 |
| 33233 | * | -0.254 |
| 33323 | * | -0.099 |
| 33332 | * | -0.247 |
| **Values for most severe EQ-5D-5L health states** | | |
| 45555 | -0.137 | N/A |
| 54555 | -0.188 | N/A |
| 55455 | -0.231 | N/A |
| 55545 | -0.103 | N/A |
| 55554 | -0.215 | N/A |
| **Value for 22222** | 0.783 | 0.72 |
| **Value for 44444** | 0.418 | N/A |
| **Minimum value (health state)** | -0.323 (55555) | -0.399 (33333) |
| **Health states ≥ 0.8, n(%)** | 224 (7.2%) | 25 (10.3%) |
| **Health states worse than dead (<0), n (%)** | 42 (1.34%) | 22 (9%) |
| **Mean value (SD)** | 0.515 (0.212) | 0.430 (0.282) |
| **Median value (Q1 - Q3)** | 0.535  (0.377 - 0.676) | 0.458  (0.252 - 0.652) |

TTO, time trade-off; DCE, discrete choice experiment; PD, pain discomfort; MO, mobility; SC, self-care; AD, anxiety depression; UA, usual activities; Q1, first quartile; Q3, third quartile; RO, Romanian; N/A, not applicable

* not displayed given that equivalent EQ-5D-5L health states might not reflect the same underlying problems
